# Combining the theory of change and realist evaluation approaches to elicit an initial program theory of the MomConnect program in South Africa

**DOI:** 10.1186/s12874-020-01164-y

**Published:** 2020-11-26

**Authors:** Eveline M Kabongo, Ferdinand C. Mukumbang, Peter Delobelle, Edward Nicol

**Affiliations:** 1 Division of Health Systems and Public Health, Stellenbosch University, Cape Town, South Africa; 2 South African Medical Research Council, Cape Town, South Africa; 3 School of Public Health, University of the Western Cape, Cape Town, South Africa; 4 Department of Public Health, Vrije Universiteit Brussel, Brussels, Belgium

**Keywords:** mHealth, MomConnect, Theory of change, Realist evaluation, Maternal and child health

## Abstract

**Background:**

One of the Sustainable Development Goals is to reduce the global maternal mortality ratio to less than 70 per 100,000 live births by 2030. In South Africa, the flagship National Department of Health MomConnect program was launched in 2014 to strengthen the quality of maternal and child health (MCH) services and improve mortality outcomes. MomConnect was rapidly rolled out with a limited understanding of how and why the program was expected to work even though studies had shown the effectiveness of the MomConnect program in improving the uptake of MCH services. This study aimed to unearth the initial program theory of the MomConnect program based on explicit and implicit assumptions of how the program was organized and expected to work.

**Methods:**

We conducted a document analysis using design- and implementation-related documents of the MomConnect program guided by the principles of Theory of Change (ToC) and Realist Evaluation (RE). Content and thematic analysis approaches were deductively applied to analyze the documents toward constructing ToC and RE-informed models. Abductive thinking and retroduction were further applied to the realist-informed approach to link program context, mechanisms, and outcomes to construct the initial program theory.

**Results:**

ToC and RE-informed models illustrated how the MomConnect program was organized and expected to work. The process of constructing the ToC provided the platform for the development of the initial program theory, which identified three critical elements: (1) the central modalities of the MomConnect program; (2) the intended outcomes; and (3) the tentative causal links indicating, in a stepwise manner of, how the outcomes were intended to be achieved. The RE approach ‘enhanced’ the causal links by identifying relevant programmatic contexts and linking the postulated mechanisms of action (empowerment, encouragement, motivation, and knowledge acquisition) to program outcomes.

**Conclusion:**

The application of ToC and RE provided an explicitly cumulative approach to knowledge generation in unveiling the initial program theory of MomConnect rather than delivering answers to questions of program effectiveness.

## Background

Target 3.1 of the Sustainable Development Goals (SDGs) aims to reduce the global maternal mortality ratio (MMR), defined as the number of maternal deaths attributed to pregnancy-related complications per 100,000 live births, to less than 70 deaths per 100,000 live births by the year 2030 [1]. Achieving this ambitious goal will require a concerted effort from respective country health authorities due to the numerous challenges impeding the prevention of maternal mortality [2].

With an institutional MMR of 133.3% per 100,000 live births in 2013/2014 [3] and 105.9 in 2018/2019 [4], South Africa (SA) has made notable progress in reducing its MMR. Although SA failed to achieve the Millennium Development Goals (MDG) 4a and 5a of reducing under-five mortality by two-thirds and maternal mortality by three-quarters between 1990 and 2015, significant progress was observed [5]. However, this figure is still high and far from the SDG target. The high MMR in SA is in part attributed to the lack of adequate maternal health care complemented by underlying social determinants of poor nutrition and poverty [6, 7].

Although SA’s MMR has improved from 2014 to 2019, complications of hypertension in pregnancy and obstetric hemorrhaging are still the main issues causing maternal mortality, which can be prevented through early uptake of antenatal care (ANC) and postnatal care (PNC) services [8]. The first ANC visit allows for easy detection of pregnant women requiring special attention, referral, and more ANC visits. Early ANC visits are also associated with adherence to antiretroviral therapy during pregnancy among HIV positive women, which reduces the chances of mother to child transmission [9]. Data from SA show that the rate of ANC visits within 20 weeks of gestation was 44.0% in 2012/2013 [9], 53.8% in 2014, and 68.1% 2018/2019 with a different point percent of 14.3 since 2014 [4]. PNC visits within the first 6 days of delivery’ were 73.0% in (2013/2014) [10].

In keeping with the SDG 3 target, SA developed a strategic plan to reduce maternal and child mortality by improving the uptake of maternal and child (MCH) services. To achieve this goal, mobile health technology (mHealth) was integrated into the healthcare system as a strategy to overcome barriers to universal health coverage and promote MCH [11]. The National Department of Health (NDoH) in 2014 launched the flagship MomConnect program to strengthen the quality of MCH services and improve mortality outcomes [12]. The MomConnect program was specifically adopted to address the peripartum factors perpetuating the high maternal and infant mortality rates in SA [12].

The MomConnect program is a digital health communication program [13], which was informed by lessons, experiences, and successes of the MAMA SA program [14, 15]. MAMA SA focused on the prevention of mother-to-child transmission (PMTCT) of HIV and consisted of a multi-channel mHealth approach to communicate healthy pregnancy and newborn child support including mobile phone-based chats and voice messages [16].

The MomConnect program was also guided by an emergent body of work around the use of mobile phones to enhance MCH programs [16–21]. Evidence suggests that communicating with pregnant women and new mothers via regular SMS and/or voice-based messages supports the adoption of healthy behaviors and increases the uptake of health services [12]. The MomConnect program leveraged elements from the MAMA SA program based on research to determine the content that would be provided in combination with the NDoH’s messaging on MCH and MCH services.

The program aimed at (i) registering all pregnant women using the public health sector; (ii) sending targeted health promotion messages during pregnancy and up until the first year of age to encourage healthy and health-seeking behavior [14]; and (iii) providing pregnant women with a feedback platform to rate the services they received [18]. MomConnect is a two-way communication channel, providing women the opportunity to rate the MCH services received at the clinics and ask questions regarding their health and that of their babies.

Although no study has shown the effectiveness of the MomConnect program in improving the uptake of MCH services, there is evidence that the national ANC visits within 20 weeks of gestation rate have been increasing in the 3 years following the implementation of MomConnect [4, 10]. Even though MomConnect has a framework for explaining how the program is expected to contribute to expected outcomes, the mechanisms underlying and health systems context that influence the outcome of the MomConnect program remain elusive. Despite this limited understanding, MomConnect was rapidly rolled out. An important step in improving the impact of MomConnect is by unearthing its program theory – the explicit and implicit assumptions of how the program is expected to work.

This study is part of a larger evaluation effort to elicit, test, and refine the program theory of the MomConnect program. The larger study has three phases: Phase 1 will focus on eliciting the initial program theory using three different approaches: a scoping review, document review, and stakeholders’ interviews. Phase 2 will be based on testing the initial program theory, while Phase 3 will entail refining the initial program theory [22]. This paper is part of Phase 1 whereby we sought to glean the initial program theory of MomConnect based on analysis of program-related documents using a combined Theory of Change (ToC) and Realist Evaluation (RE) approaches.

### Methodological approaches

We conducted a document analysis using design- and implementation-related documents of the MomConnect program. Document analysis is a systematic procedure for reviewing or evaluating both printed and electronic (computer-based and Internet-transmitted) material [23]. Our document analysis was informed by the ToC and RE approaches [24].

ToC and RE are members of the theory-driven evaluation (TDE) or theory-based evaluation (TBE) family. TDE/TBE are regarded as key to untangling the multiple processes between policy intent and outcomes by investigating program implementation, and the causal link processes that trigger outcomes [25, 26]. Traditionally, ToC and RE are applied separately in different studies to obtain corresponding outputs. However, we considered the two approaches together in our study to obtain a more robust initial program theory for the MomConnect program.

According to Taplin et al. [27], ToC makes underlying assumptions explicit by delineating the different components: input, outputs, outcomes, and impact [28, 29]. ToC can be viewed as both a product and process [27]. As a product, its inquiry results in specific outcomes in a narrative and/or visual form and offers a framework for sense-making that needs to be used, revisited, and adapted as the project or program progresses. As a process, the ToC model shows how a goal will be reached [27].

**Fig. 1:**
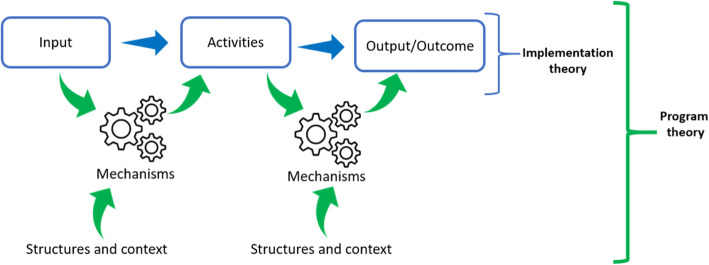
Relationship between implementation theory and program theory. Adapted from Eastwood et al., [31]

While ToC illustrates ‘how’ change occurs through program implementation, RE goes deeper by establishing ‘why’ through a mechanism-based generative causal framework. Blamey and Mackenzie [30] propose that the ToC can be used as a means of explicating intervention implementation theory for program planning, improvement, and the development of robust monitoring systems at a whole program level; while RE can be used to examine, in detail, how and why the different aspects of the program leads to the observed or intended outcome – program theory (Fig. 1).

RE focuses on providing a ‘causation-based explanation’ – depiction of cause-effect relationships among elements [24] – to provide explanations of program effectiveness [32]. RE is a theory-driven approach to evaluation informed by principles of critical realism – social structures and agents having underlying causal powers that interact to cause an observed behavior [33]. RE aims to unearth theories to explain how and why the intervention is supposed to trigger change [34]. RE theories are formulated by conceptualizing the relationship between the context (C) within which the program is implemented, the generative mechanisms of change (M), and the observed outcomes (O). This conceptualization is achieved by formulating the Context-Mechanisms-Outcome configurations (CMOs) [35]. Of importance, nevertheless, is the notion that the Intervention (I) must be accepted by the users, Actors (A), for it to be successful. Therefore, we considered adding elements of the Actors (A) and intervention (I) modalities to the original CMOs heuristic tool and adopted the intervention-context-actor-mechanism-outcomes (ICAMO) configuration [33].

There are multiple definitions of ‘mechanism’. In RE terms, a mechanism (M) relates to a combination of intended and unintended resources provided by the program and the response to those resources by stakeholders [36]. A mechanism is also defined as the reasoning and responses that participants attribute to the resources, opportunities, and constraints of a program to bring about the observed outcomes [37, 38]. Others have defined mechanisms as the underlying entities, process, or structures, which operate in a particular context to generate outcomes of interest [38]. Context (C) is the physical or perceived conditions, which allow the mechanisms to come into operation or remain inactive. The context can also be defined as circumstances that configure the settings in which the intervention takes place, and the action sets of activities that will trigger the change [24]. An outcome (O), which can be represented in short and intermediate terms, is what is observed resulting from the interaction between the context and mechanism that is triggered by the intervention, and could be measured as the impact of the intervention [39].

**Fig. 2:**
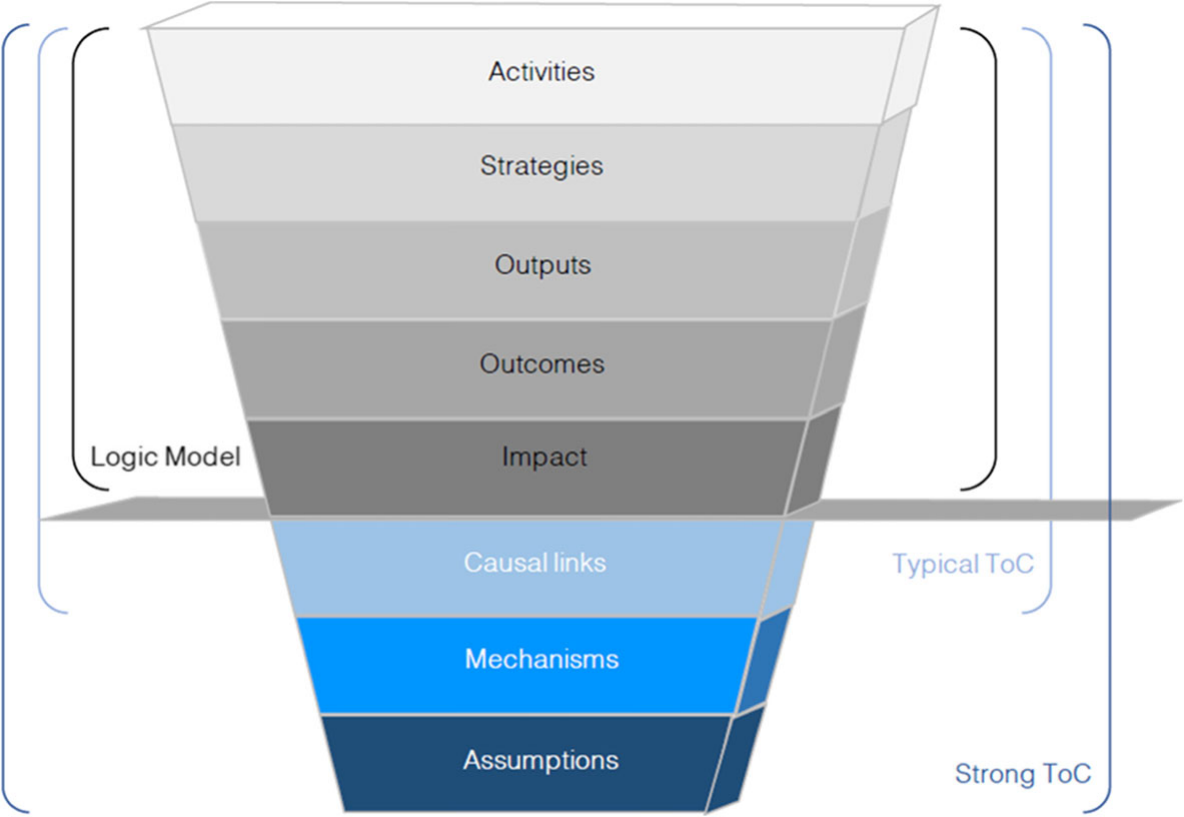
A framework illustrating the features of ToC and RE (Dhillon and Vaca [24])

By combining the ToC and RE approaches we intended to unearth, in a systematic manner, the assumptions underlying the MomConnect program and speculate the changes likely to take place as a result of the intervention [40]. Our intention to glean the initial program theory of the MomConnect program informed by ToC and RE approaches was based on harnessing the overlapping and complementary features of these two approaches. To this end, we adopted a framework proposed by Dhillon and Vaca [24] (Fig. 2) integrating the features of both ToC and RE approaches and how they complement each other to inform the elicitation of a robust and testable program theory.

## Methods

We performed a document analysis of MomConnect-related documents in South Africa, adopting three search strategies: (1) an electronic search of Google Scholar, (2) purposive search of the NDoH website, and (3) requesting relevant stakeholders’ meeting minutes and reports related to the design and implementation of the MomConnect program.

The Google Scholar search covered the period 2014–2019, i.e. from the year that MomConnect was implemented until the year the document review was initiated. Using the terms ‘MomConnect intervention’ OR ‘MomConnect program’ resulted in 266 hits. Combining the terms (‘MAMA South Africa’ AND ‘MomConnect program’) resulted in 86 additional documents, resulting in 352 documents. Eighteen documents relating to the MomConnect program were found by searching the NDoH website. Ten meeting minutes and reports were retrieved from key stakeholders resulting in a total of 380 documents (Table 1).

**Table 1:** Document sources and numbers obtained

Source or document	Number
Google search using ‘MomConnect intervention’ or ‘MomConnect program’	266
Google search using ‘MAMA south Africa and MomConnect program’	86
Browsing the NDoH website	18
Requesting MomConnect program design and implementation minutes and reports	10
Total	380

**Fig. 3:**
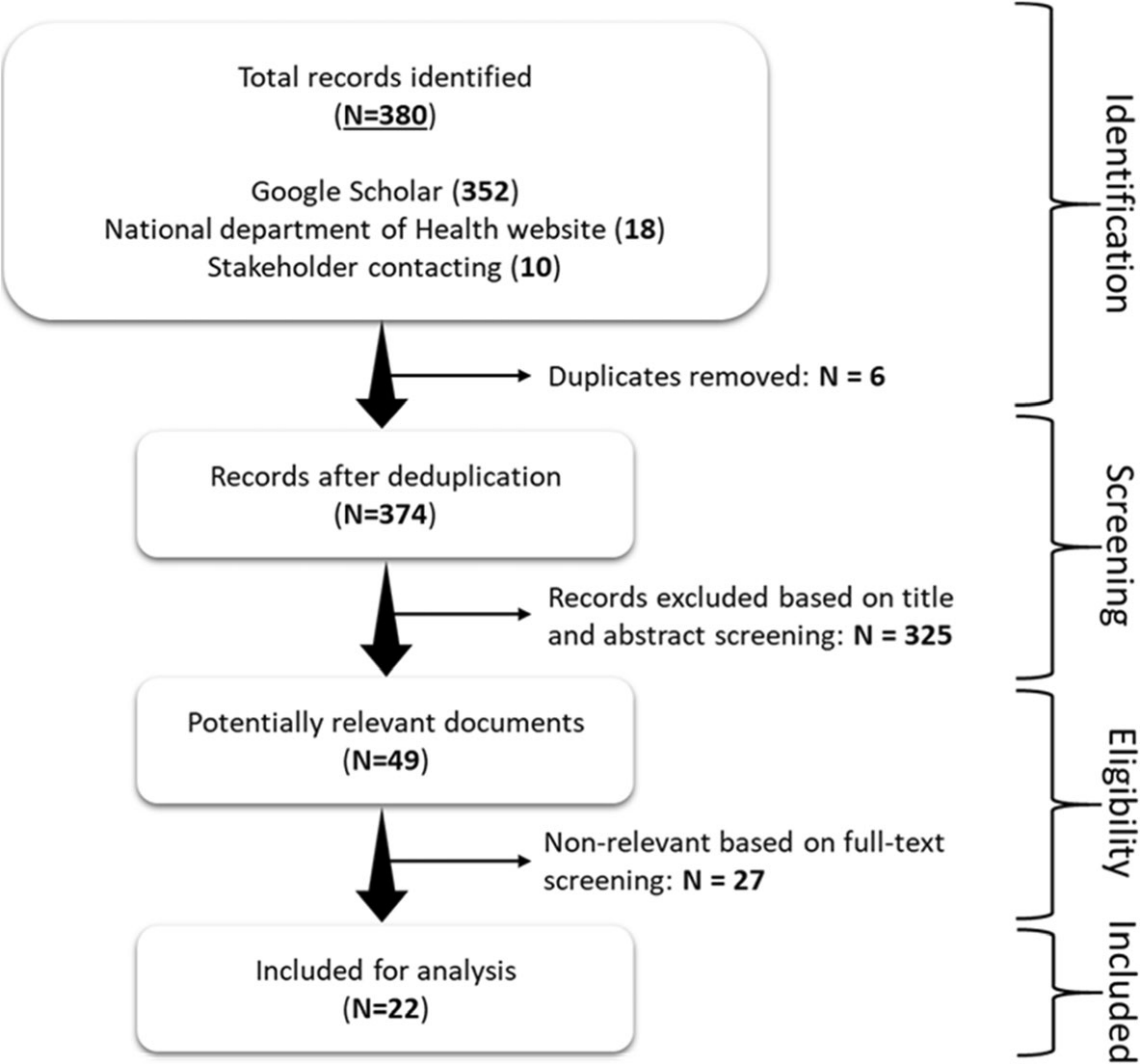
Document selection flowchart

After screening by title and abstract, 358 documents and duplicates were removed. Screening of the documents for eligibility hence identified 22 documents, which were included in the final analysis (Fig. 3). The characteristics of the selected documents are summarized in Additional file 1**.**

### Data analysis

Our data analysis involved skimming (superficial examination), reading (thorough examination), and identifying themes within the documents associated with the elements of ToC and the concepts of RE theory formulation. This iterative process combined elements of content and thematic analyses [23]. Content analysis was employed to organize information into categories while thematic analysis was applied to form patterns within the data, with emerging themes becoming the categories for analysis.

Inductive coding and deductive categorizing were performed to uncover and align themes related to the ToC framework (Fig. 1). After obtaining the ToC, further data coding was applied to identify statements or portions of the texts attributable to the constructs of mechanism and contexts. Abduction – inventive thinking required to imagine the existence of mechanisms and retroductive thinking – a mechanism-focused approach to inference making, which seeks to clarify the basic prerequisite or conditions of a phenomenon, were applied to explore and link elements of intervention modalities, context, actors, mechanisms, and outcomes to obtain the ICAMO configuration.

## Results

**Fig. 4:**
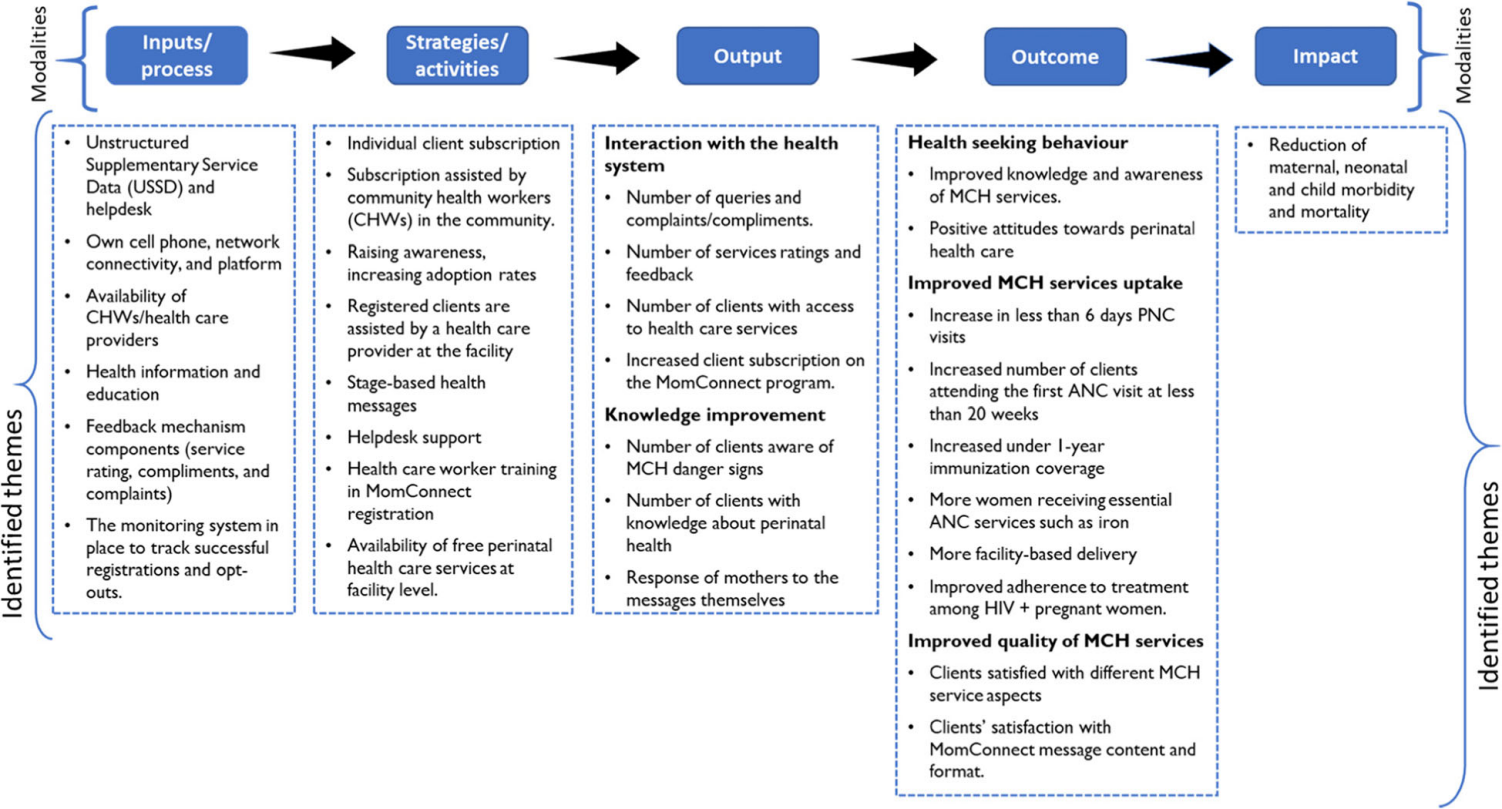
Identified themes based on Theory of Change framework

Figure 4 presents a summary of the themes obtained from the content and thematic analyses. The impact was identified as reductions in maternal, neonatal, and child morbidity and mortality. Outcomes were related to health-seeking behavior; improved MCH services uptake, and improved quality of MCH services. The output themes speak to interactions with the health system and knowledge acquisition.

### MomConnect program theory (ToC)

**Fig. 5:**
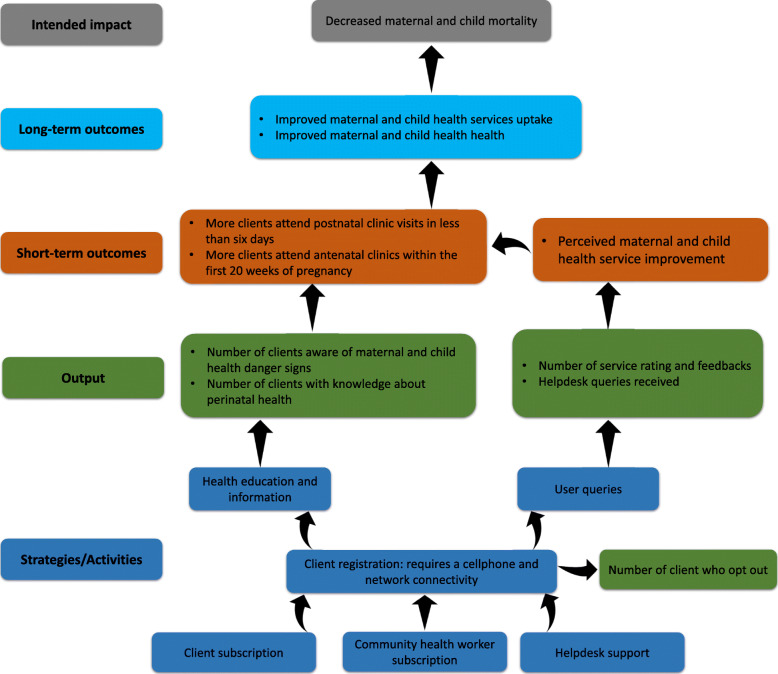
The MomConnect program theory of change

The process of constructing the ToC provided three critical features toward developing an initial program theory of the MomConnect program. Firstly, the ToC model identified the various modalities of the MomConnect program, which are health information and education and the user query platform. Secondly, it clearly articulated the intended outcomes (short-term and long-term) of the MomConnect program. Finally, the ToC provided tentative causal links in a stepwise manner to show how the outcome is to be achieved (Fig. 5).

### The realist evaluation-informed model

Our ToC underpinned the process of developing the initial program theory, which is based on the RE methodological approach. As highlighted in Fig. 1, additional elements are required to make the shift from a ToC to a realist program theory. Notably, the identification of mechanisms and contextual elements are critical to establishing a generative causality – how the intervention modalities are interpreted and acted upon by the actors (mechanisms) and under what conditions (context) leading to the hypothesized impact. Table 2 illustrates a thematic analysis of the data based on RE principles and the ICAMO heuristic tool.

**Table 2:** Intervention, context, actors, mechanism and outcome elements of realist evaluation

Modalities	Identified themes
**Intervention (I)**	- Stage-based health information up to a child’s first birthday - Interaction with the health system (service ratings and feedback)
**Context (C)**	**Health systems context** - Political clout: NDOH leadership and ownership of MomConnect - Awareness-raising **Facility responsiveness** - Staff training sessions on helpdesk, registration procedures and telephonic support - Staff adoption and adaptation of the new technology - Staff availability **Intervention-related context** - Anonymous nature of the SMS helpline - Network availability & ownership of a mobile phone - Length of the registration process
**Actors (A)**	- Pregnant women - Mothers of newborns - Community health workers - Health care providers
**Mechanism (M)**	- Encouragement - Empowerment - Motivation - Knowledge acquisition
**Outcome (O)**	- (Perceived) improvement in quality of MCH services - Improved health-seeking behavior and MCH services uptake

**Fig. 6:**
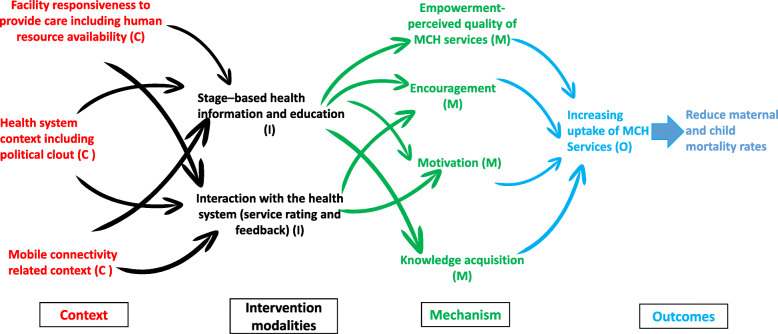
Realist evaluation model for MomConnect program

Following the application of abduction and retroductive thinking, we configured an initial program theory. Table 2 presents the thematically obtained elements used to develop the RE model (Fig. 6), which links the Intervention (I), context (C), causal pathways of outcomes (O), and the mechanisms (M) describing the causal chains, or causal pathways that explain how, when and/or why the MomConnect program messages trigger the expected results.

A key component of the program consists of sending pregnancy-related stage-based messages (I) to pregnant women and mothers (A), facilitated by health systems conditions such as facility readiness to provide care (C), and intervention-related resources and mobile connectivity (C). The pregnancy-related stage-based information was envisaged to encourage and empower (M) users through knowledge acquisition to motivate them (M) thus improving their health-seeking behavior (O).

Interaction with the health system (service rating and feedback) (I) is influenced by health system conditions such as facility readiness to provide care (C) and health care worker availability and buy-in (C). By rating the services received at the facility level, women and mothers are expected to feel empowered (M), which in turn would result in improved quality of health care delivery (O) and improved uptake of MCH services (O).

## Discussion

We aimed to glean an initial program theory of the MomConnect initiative to understand how and why the program works (or not). We used the ToC and RE approaches to elicit the initial program theory [41]. Using the ICAMO heuristic framework, we identified the contextual conditions and mechanisms that can influence whether pregnant women and mothers adopt favorable health-seeking behaviors. Our study identified three main contextual conditions – facility responsiveness to provide care including human resource availability, health system context such as political clout, and mobile connectivity related context – and four causal mechanisms – empowerment and perceived quality of MCH services, encouragements, motivation, and knowledge acquisition.

Findings from the study revealed that if pregnant women and mothers receive pregnancy related information by ‘SMSs’, they will become sensitized about existing MCH services tailored to the stage of their pregnancy. Being sensitized can encourage and motivate them to attend clinic appointments, ultimately decreasing maternal and child mortality. We also found that the interaction with the health system (service rating and feedback) provided by the MomConnect program is likely to empower users, since they feel that it can improve the quality of services offered at healthcare facilities, thus improving the perceived quality of MCH services and, in turn, the uptake of health services.

These findings are consistent with the work by Ruton et al. [42] in which CHWs were given mobile phones to report data on MCH indicators using text messages (Rapid SMS a two-way communication). The reminders for clinic appointments on ANC, the probable delivery date, and PNC were generated automatically. These reminders were sent to the CHWs who in turn contact the clients, resulting in an increase in routine care attendance and facility deliveries. RapidSMS influenced the use of MCH services, whereby an additional support package was provided such as training of health care providers (HCPs), equipment, and supervision [42]. Our finding is also consistent with the mHealth ImTeCHO intervention in India [43], which established a significant relationship between the use of mobile phone technology by HCPs and changes in the working environment. Training, supervision, workload, and remuneration motivated HCPs to perform well in their duties.

Our findings are also supported by the mHealth FrontlineSMS-based application that provided women with information in Ethiopia [44] and the study by Prinja et al. in India [45], which used a ToC approach to explain how the use of mHealth supported and improved the quality of counseling delivered by HCPs. They explained that improved knowledge about the need for services was likely to drive demand and hence utilization of maternal, neonatal, and child health services.

A study conducted by Hategeka et al. [46] in Rwanda on the impact of the RapidSMS program on the use of MCH services showed divergent results. The lack of impact of RapidSMS on ANC services uptake was attributed to the infidelity in the implementation of the program. The study conducted in Rwanda by Musabyimana et al. [47] on implementers’ and community health workers’ experiences of the RapidSMS program also identified challenges to the successful implementation of the program, which included lack of motivation and incentives, high turnover rates of CHWs, and lack of regular CHW training. These findings are indicative of important contextual elements that can influence the success of the MomConnect program.

Our initial program theory is consistent with some existing models such as the Elaboration likelihood model (ELM) and the extensive Elaboration likelihood model (eELM) [48], and the Theoretical Domains Framework (TDF) [49] in behavior change. ELM and eELM by Adam et al. [48] focused on Mobile Video intervention, which stipulates that the changes in attitude that predict a desired behavioral outcome such as exclusive breastfeeding can be explained in two pathways. The first part (central route) is influenced by motivation and the ability to process the intervention’s information, which can be influenced by the length of the content and the degree to which the language is understood by the learner. The second part (peripheral route) relies on clues embedded in the information delivery method, which contributes to its relative acceptability by the user. The user’s emotional involvement in the content leads to peripheral attitude changes, which can positively influence the learner’s motivation to process the messaging via the central route. Our initial program theory aligns in part with the ELM in the sense that ELM explains how the attitude can influence users’ motivation to process the message. The empowerment and encouragement derived from MomConnect’s messages allow clients to become motivated toward perinatal health care, which in turn improves the uptake of MCH services.

The Theoretical Domains Framework (TDF) was developed to assess implementation problems, support intervention design, and investigate the fidelity of intervention delivery [49]. TDF posits that a good understanding of psychological capability and reflective motivational processes in knowledge, skills, social/professional role and identity, beliefs about capabilities, optimism, beliefs about consequences, reinforcement, intentions, goals and memory, attention and decision processes can influence behavior. TDF explains that the social influences and environmental context and resource domains point to organizational and systems context for change [49]. The TDF supports our model in the sense that, some of the TDF domains were included in our model, such as knowledge acquisition, which is a central modality provided by MomConnect messages through health education and information (I). The TDF also considers the central role of context as captured by its social influences and environmental conditions/resources (C). The TDF also considers the notion of the ‘mechanism of action’ in the domain of beliefs about capabilities and emotion (M). Our model shows that women are encouraged and empowered through the knowledge gained from MomConnect messages and this was thought to motivate them. Our model differs from the TDF in that the TDF offers little conceptual configurations (causal links) between the elements in their different domains.

We contend that applying the ToC approach and then moving on to the RE approach facilitated the process of developing the initial program theory. The ToC was used to explicate the implementation theory for program planning, improvement, and the development of robust monitoring systems at a macro program level [30]. The RE approach was then used on a more micro-level (user-oriented understanding) of how and why the intervention would work or not. The benefit of combining the ToC and RE is shown by the fact that ToC makes it easier to trace causes if outcomes are not achieved at the management level. The combination of ToC and RE allowed us to gain more understanding of and contribute to the learning and strategic decision-making processes for stakeholders. Also, the merging of these two approaches promote a better understanding of the successes and challenges of the MomConnect program. Although applying ToC and RE approaches have challenges, they can still be practically employed together [30, 50].

### Rigor and trustworthiness

The formulation of the ToC and initial program theory was achieved in an iterative process – moving back and forth between the data, the ToC, and developing the initial program theory. All authors held discursive weekly meetings to conceptualize and finalize the ToC and initial program theory.

### Strengths and limitation

Overall, our study has established theoretical foundations and comprehensive requirements for the development of program theory in mHealth interventions (MomConnect), to support decision making, and decision support capabilities. Our study contributes to the cumulative theory development activities, mHealth, MCH services, and decision support. The program theory and connection between mHealth, improved MCH services, and decision making is highlighted in our ToC and RE, and the implications for developers of mobile-based interventions are that the model provides a holistic approach to system thinking, systems analysis, and design. This assisted in actualizing the next phases of our study, which is the development of a program theory using a scoping review and stakeholders’ interviews.

The study has several limitations as both ToC and RE have their challenges. For instance, the ToC model tends to be linear and lacking detail, particularly around causality. The model can be too descriptive and ignore issues of power and conflicting theories. ToC is also time and resource-intensive and has some difficulties in dealing with multiple perspectives, unexpected outcomes, and critical analysis. In RE, on the other hand, it can be difficult to identify and conceptualize mechanisms and separate context from mechanisms. RE lacks substantive theory in some fields, multiple and conflicting theories in others, and uses inaccessible jargon. Like ToC, RE is also time and resource-intensive. A final limitation is that this study only included document reviews and excluded studies such as RCTs and systematic reviews.

## Conclusion

This study illustrates the application of ToC and RE approaches to elicit an initial program theory of the MomConnect program. Because both approaches take an explicitly cumulative approach to knowledge generation, they have been usually applied in isolation. Based on the experience from our study, we concur that ToC and RE approaches can be practically employed together and that their synergism can partially overcome the critiques of each methodology. We found that the application of both ToC and RE enhances learning and the unveiling of the initial program theory rather than delivering answers to questions of program effectiveness.

## Supplementary Information

**Additional file 1 **List of documents containing clauses for MomConnect program (*n* = 22) the Document was ordered by date of publication.

## Data Availability

All data generated or analyzed during this study are included in this published article and its supplementary information files - Additional file 1.

## Abbreviations

ANC: Antenatal care
ART: Antiretroviral therapy
CHWs: Community Health Workers
CMOc: Context Mechanism Outcomes configuration
ELM: Elaboration likelihood model
eElm: Extensive Elaboration likelihood model
HCP: Health Care Providers
ICAMO: Intervention Context Actors Mechanism Outcomes configuration
MCH: Maternal and Child Health
MMR: Maternal Mortality Ratio
MNCH: Maternal, Neonatal, and Child Health
NDoH: National Department of Health
PMTCT: Prevention of mother-to-child transmission of HIV
PNC: Post Natal Care
PT: Program theory
RCTs: Randomized Controlled Trials
RE: Realist Evaluation
SA: South Africa
SDGs: Sustainable Development Goals
TBE: Theory-based evaluation
TDE: Theory-driven evaluation
TDF: Theoretical Domains Framework
ToC: Theory of Change
USSD: Unstructured Supplementary Service Data
WHO: World Health Organization
