# Supplementary material for: Combining the theory of change and realist evaluation approaches to elicit an initial program theory of the MomConnect program in South Africa
Source: BMC Med Res Methodol. 2020 Nov 26;20:282. doi: 10.1186/s12874-020-01164-y (PMC7691101; doi:10.1186/s12874-020-01164-y)
Supplement: Supplementary file 1 — Additional file 1 List of documents containing clauses for MomConnect program (n = 22) the Document was ordered by date of publication. [file 12874_2020_1164_MOESM1_ESM.docx]

Additional file 1: List of documents containing clauses for MomConnect programme (n=22) the Document was ordered by date of publication

|  | **Title** | **Author/year** | **Description** |
| --- | --- | --- | --- |
| DR1 | MomConnect: one year of operation case study [1] | National Department of Health, South Africa / 2015 | This document describes how the MomConnect was conceived and how all started. It is explaining the global context of maternal health in South Africa. The first point of this document provides the insight on how the mHealth can make a difference. The second discussed the South African’s maternal and child health (MCH) obligations and other mHealth projects in existence at that time. Also, the document discussed how education can empower pregnant women and mothers and results in improving MCH health and health services utilisation. Another discussion was on the MomConnect as the first large scale mHealth intervention that is supported by the national and provincial health authorities. Also, explained how pregnant women and mothers are empowered to report good and bad services received at health facilities. It also explains the future opportunities of the MomConnect. |
| DR2 | MomConnect: Launching a National Digital Health Program in South Africa [2] | NDoH, South Africa /2014 | This document explain how the MomConnect was developed and implemented by outlining the design process and the challenges of how to lower the maternal and child mortality. It described the challenges of the MomConnect as a programme that can be scalable, targeted and trackable. |
| DR3 | Operational research for MomConnect [3] | NDoH, South Africa Technical Report/October 2015 | This document describes a technical report on the implementation of MomConnect at the facility level and how the NDoH is sought to understand why registration targets are not being achieved at the facility level. In addition, the document describes how facilities were trained on MomConnect, and whether there were common characteristics between high and low registration facilities. It described the process evaluation elements and their relation to MomConnect and an example of low and higher performed facility. Process evaluations assess the implementation of the MomConnet programs and help provide explanations for it successes or failures. It explains that conducting a process evaluation for formative purposes during the early implementation stages of a program allows for program improvement by identifying inadequacies during various program stages; from the design, planning and training to actual implementation. |
| DR4 | Market Research Report: Experience of Mothers Using MomConnect [4]. | NDoH, South Africa  9 October 2015 | This document was prepared for the Department of Health by the Praekelt Foundation in support of the NDoH “MomConnect” project. It describes the response rate and completion of the MomConnect programme by describing the achievement that the MomConnect was able to achieve during one year since the implementation. It discussed a series of surveys conducted with mothers who are actively using the MomConnect service to understand the factors that influence whether mothers convert from the set of basic Public messages to the full set of stage-based Clinic messages, and the response of mothers to the messages themselves, including the perceived value and helpfulness of the messages. |
| DR5 | Using mobile technology to improve maternal, child and youth health and treatment of HIV patients Africa [5] | NDoH, South January 2016 | This document describes the cellphone penetration in Africa and how does MomConnect work? The evidence for mobile messaging to mothers and the vision for future. |
| DR6 | Taking digital health innovation to scale in South Africa: ten lessons from MomConnect [6] | Peter et al. 2018 | The purpose of this document was to summarise lessons learnt across the domains of leadership and partnerships, technology and architecture, content and user engagement, financial health, and monitoring and evaluation. It provides same selected lessons learned from the MomConnect and how it may provide insights to other digital health programmes. In addition, the document describe how framework provided by WHO’s ‘mHealth assessment and planning for Scale’ toolkit is used to summarise the lessons learnt. |
| DR7 | Digital health vision: could  MomConnect provide a pragmatic starting point for achieving universal health coverage in S. Africa and elsewhere?[7] | Mehl et al., / 2018 | This document is about what MomConnect can offer as a national vision and architecture for digital health. It describes opportunities to enhance MomConnect and explain how the MomConnect demonstrates an extensible and modular digital approach for Universal Health Coverage (UHC0 goals across Africa. It shows how MomConnect can allow moving from universal pregnancy registration to Universal Health Coverage |
| DR8 | Mobile health messaging service and helpdesk for S African mothers  (MomConnect): history, successes and challenges [8] | Barron et al./ 2018 | This paper article discussed the history of mHealth messaging services in South Africa, and the designing of the MomConnect service. Also, it described the MomConnect programme successes, MomConnect's limitations and implementation challenges, and vision for the future. |
| DR9 | Achieving scale, sustainability and impact: a donor perspective on a mobile health messaging service and help desk (MomConnect) for S. African mothers[9] | Peter /2018 | This document describes the Scale, sustainability and impact of the MomConnect. It discussed the MomConnect programme and the role of public–private partnership. |
| DR10 | Designing for scale: optimising the health information system architecture for mobile maternal health messaging in South Africa (MomConnect) [10] | Seebregts et al./2018 | This document described in details the design and the implementation of the MomConnect programme by describing the architecture of the MomConnect technical platform, planned as a nationally scalable and extensible initiative. It provides technical considerations in resource-constrained environments, building out the technical infrastructure. Also, it discussed how to get data into the system. |
| DR11 | The MomConnect helpdesk: how an interactive mobile messaging programme is used by mothers’ in S Africa [11] | Xiong et al., /2018 | This document described the MomConnect help disk, the reason for health desk use and it utilisation. |
| DR12 | Unpacking the performance of a mobile health information messaging program for mothers (MomConnect) in South Africa: *evidence on program reach and messaging exposure* [12] | Lefevre et al., /2018 | The document described what is the MomConnect and measuring exposure to maternal health information messages and discussed the optimal pathway for pregnant women from point of contact with the health system, to registration into MomConnect, message delivery and receipt and the intended effects on behaviour. |
| DR13 | mHealth compendium special edition 2016: reaching scale [13] | African Strategies for Health (ASH) project in collaboration with USAID /AFR./ 2016 | This document discusses the promoting maternal and child health in South Africa through mobile health messaging and feedback on services received the contribution of the mHealth in improving the health status of populations across Africa through identification of and advocacy for best practices, enhancing technical capacity, and engaging African regional institutions to address health issues in a sustainable manner. |
| DR14  hands | Report on pretesting of MomConnect short messaging Services (SMS) | **Report**  Soul city Institute health education and development communication | This document discussed the pre-test on the MomConnect SMS. It assesses whether the target audience understand the SMSs, how appropriate they are and the relevance of the SMSs for use in facilitating communication around issues of pregnancy and baby’s first year of life; as well as to find out the extent to which these messages can spark conversation and information seeking behaviour among pregnant women and new mothers in South Africa.  It described the MomConnect process development, to assess whether the target audience understand the SMSs, how appropriate they are and the relevance of the SMSs for use in facilitating communication around issues of pregnancy and baby’s first year of life; as well as to find out the extent to which these messages can spark conversation and information seeking behaviour among pregnant women and new mothers in South Africa. |
| DR15 | USSD Service Rating through the South African National Department of Health’s MomConnect Project: *Citizen based monitoring of Antenatal Clinics* | MomConnect registration possess | The document described in details the service rating feedback mechanism that allows women to provide quantitative feedback on their experiences at the clinic and how the feedback can potentially be used to get managers to address issues and highlight good performance. It details the MomConnect registration process and how it uses a mobile phone-based technology called Unstructured Supplementary Service Data (USSD). It provides a basic analysis of the national and provincial level data and show how it can be utilised to effectively monitor patient-oriented service. |
| DR16 | Knowledge and attitudes regarding subscription to mobile health messaging (mHealth) for pregnancy care and infant health | **Report**  Bradley et all 2014 (UCT) | This report strived to establish the knowledge and attitudes of pregnant women and mothers with infants regarding subscription to mobile health messaging for maternal and infant health |
| DR17 | MomConnect National Monitoring & Evaluation 1st Quarterly Report (April 2014 – March 2015) | **Report**  A partnership between the university of stellenbosch / university of the western cape / NDoH 3/2015 | The document a M&E of the MomConnect which is the effort joint by the Stellenbosch University, the University of the Western Cape, Health Information Systems Program (HISP) and the National Department of Health (NDoH). It aimed at proved the the answer to the following questions:  • Does MomConnect achieve the objective of registering all pregnant women in South Africa on a centralized database?  • Does MomConnect improve MCH health seeking behaviour /services uptake of pregnant women in South Africa?  • Does MomConnect increase the (perceived) quality of MCH services in South Africa? |
| DR18 | \| MomConnect National Monitoring & Evaluation 2nd Quarterly Report (April 2014 – August 2015) \| \| --- \| | **Report**  Stellenbosch University, and University of the Western Cape | This document described the second quarterly M&E report and provides the results of the longitudinal analysis of MomConnect programme outputs from the DHIS2/‘MomConnect’ database for the period August 2014 to the end of August 2015; and on selected programme outcomes from the DHIS1.4 database from April 2014 up till the end of June 2015. |
| DR19 | MomConnect  National Monitoring and Evaluation 3^rd^ Report January 2015 to December 2015 | **Report**  Stellenbosch University, and University of the Western Cape | This document report describes the service rating that enable women to provide feedback on their satisfaction with the service they received at the clinic with regards to respect for privacy, cleanliness, friendliness, waiting time feel and waiting time length. It assess the usefulness of the data at facility level to identify deficiencies in service delivery to inform actions to improve the quality of care. |
| DR20 | MomConnect National Monitoring & Evaluation 4th Quarterly Report (April 2014 – December 2016) | **Report**  A partnership between the university of Stellenbosch / university of the western cape / NDoH Report, 31 May 2016 | This document is a fourth quarterly MomConnect evaluation report that provides an analysis of trends in selected DHIS 1.4 outcome indicators to respond to questions raised by the NDOH. In particular it sought, i) to understand if any changes were evident in busier ‘high volume’ ANC facilities (previous analysis of all facilities had demonstrated little effect of MomConnect); ii) to compare the outcomes between ‘high volume’ health facilities with high (>80% of clients) and low (<20% of clients) MomConnect registrations; and iii) to analyse MomConnect data at facility level. |
| DR21 | Monitoring & Evaluation Final Report  18 April 2017 | **Report**  Stellenbosch University, and University of the Western Cape | The primary objective of this Monitoring and Evaluation study was to assess whether MomConnect achieved its objective of registering all pregnant women in the public health sector in South Africa on a national database and improved the MCH health seeking behaviour of registered women. Secondary objectives included assessing MomConnect program perception and awareness among registered women and health care workers, and assessment of the perceived quality of care received by pregnant women in general. |
| DR 22 | MomConnect: an exemplar implementation of the Health Normative Standards Framework in South Africa [14]. | Seebregts et al./ 2016  SAHR 2016 | This document is a chapter that describes the development of the technical infrastructure for the MomConnect system, and the alignment of its information management component with the Health Normative Standards. The emphasis in this chapter is on describing the processes followed to implement a technical infrastructure using the HNSF model, and its construction in a modular manner in order to facilitate ongoing alignment with the evolving NDoH technical infrastructure. |

**Reference list**

[1] National Department of Health. *MomConnect one year of operation case study*, file:///C:/Users/US/Downloads/momconnectcasestudy6.pdf (2015).

[2] National Department of Health South Africa. MOMCONNECT: Launching a National Digital Health Program in South Africa. 2014; 28.

[3] National Department of Health. *OPERATIONAL RESEARCH FOR MOMCONNECT*, file:///C:/Users/US/Downloads/momconnect12oct2015.pdf (2015).

[4] National Department of Health. *Market Research Report: Experience of Mothers Using MomConnect*. 2015.

[5] Peter JE, Barron P, Pillay Y. Using mobile technology to improve maternal, child and youth health and treatment of HIV patients. *South African Med J* 2016; 106: 3.

[6] Peter J, Benjamin P, Lefevre AE, et al. Taking digital health innovation to scale in South Africa : ten lessons from MomConnect. *BMJ Glob Heal* 2018; 3: 1–4.

[7] Mehl GL, Tamrat T, Bhardwaj S, et al. Digital health vision : could MomConnect provide a pragmatic starting point for achieving universal health coverage in South Africa and elsewhere ? *BMJ Glob Heal* 2018; 3: 1–5.

[8] Barron P, Peter J, LeFevre AE, et al. Mobile health messaging service and helpdesk for South African mothers (MomConnect): history, successes and challenges. *BMJ Glob Heal* 2018; 3: e000559.

[9] Peter J. Achieving scale , sustainability and impact : a donor perspective on a mobile health messaging service and help desk ( MomConnect ) for South African mothers. *BMJ Glob Heal* 2018; 3: 1–3.

[10] Seebregts C, Dane P, Parsons AN, et al. Designing for scale : optimising the health information system architecture for mobile maternal health messaging in South Africa ( MomConnect ). *BMJ Glob* 2018; 2: 1–7.

[11] Xiong K, Kamunyori J, Sebidi J. The MomConnect helpdesk : how an interactive mobile messaging programme is used by mothers in South Africa. *BMJ Glob Heal* 2018; 3: 000578.

[12] Lefevre AE, Dane P, Copley CJ, et al. Unpacking the performance of a mobile health information messaging program for mothers ( MomConnect ) in South Africa : evidence on program reach and messaging exposure. *BMJ Glob Heal* 2018; 3: e000553.

[13] USAID. mHEALTH COMPENDIUM SPECIAL EDITION 2016: REACHING SCALE. *Am People* 2016; 1–92.

[14] Seebregts C, Barron P, Tanna G, et al. MomConnect: an exemplar implementation of the Health Normative Standards Framework in South Africa. *S Afr Heal Rev* 2016; Jan 2016: 125–135.
